# Xanthoma disséminatum sur peau noire: retard diagnostique et difficulté thérapeutique d’un cas d’évolution fatale en Afrique subsaharienne

**DOI:** 10.11604/pamj.2021.39.248.30724

**Published:** 2021-08-18

**Authors:** Mame Téné Ndiaye Diop, Babacar Niang

**Affiliations:** 1 Service de Dermatologie, Hôpital d’Enfant Albert Royer, Université Cheikh Anta Diop De Dakar, Dakar, Sénégal,; 2 Service de Pédiatrie, Endocrinologie, Hôpital d’Enfant Albert Royer, Université Cheikh Anta Diop De Dakar, Dakar, Sénégal

**Keywords:** Xanthoma disséminatum, immunohistochime, enfant, Afrique subsaharienne, peau noire, xanthoma disseminatum, immunohistochim, child, sub-Saharan Africa, black skin

## Abstract

Medical image Xanthoma disseminatum is a proliferative non-Langerhans histiocytosis first described by Montgomery in 1938. We here report the case of a 8-year-old Senegalese boy having skin type VI who died. In 2015 this boy presented with slowly progressive dermatosis, which had occurred at the age of 7 years. Dermatological examination showed diffuse xanthomatous orange papules and nodules on the trunk, axillary and inguinal folds, scrotum, neck and face. Extradermatological examination was normal. Histopathological examination of a skin biopsy showed dermal infiltrate composed of histiocytes and multinucleated giant Touton cells. The diagnosis of non-Langerhans histiocytosis was retained, subject to immunohistochemical analyses which were not availabe. Immunohistochemical analyses were too expensive in the absence of health insurance (our patient was from a low socio-economic background). Tests measuring the amount of lipids were normal. At the age of 11 years, diabetes insipidus and systemic involvement of mediastino-pulmonary and abdominal and pelvic organs supported the diagnosis of xanthoma disseminatum. Mediastino-pulmonary disease was complicated by pneumonitis with dyspnoea which didn´t regress on prednisone treatment (1mg/kg/day). The patient underwent surgical resection of a massive mediastinal mass that compressed the organs. Five months later (in 2019), the patient died due to respiratory distress. The difficulty of diagnosing non-Langerhans histiocytosis in our context is largely related to the inaccessibility of immunohistochemistry. Indeed, if CD68 positive and CD1a negative labeling cells had been early detected, fairly early diagnosis would be made. Thus, we could have offered treatments to improve the quality of child´s life.

## Image en médecine

Le xanthoma disseminatum est une histiocytose proliférative non langerhansienne, décrite par Montgomery en 1938. Nous rapportons l´observation d´un garçon sénégalais de 8 ans de phototype VI chez qui l´évolution a été fatale. Un garçon de 8 ans consultait en 2015 pour une dermatose évoluant depuis l´âge de 7 ans. L´examen dermatologique retrouvait des papulo-nodules orangés, xanthomateux diffus, sur le tronc, les plis axillaires et inguinaux, le scrotum, le cou et le visage. L´examen extra-dermatologique était normal. L´histopathologie cutanée montrait un infiltrat dermique composé d´histiocytes et de cellules géantes multinucléées de Touton. Le diagnostic d´histiocytose non Langerhansienne avait été retenu sous réserve d´une immunohistochimie non disponible. Le coût de l´immunohistochimie était excessivement cher en l´absence d´assurance maladie pour notre malade de bas niveau socio-économique. Le bilan lipidique était normal. À l´âge de 11 ans, est survenue un diabète insipide et une atteinte systémique des organes médiatino-pulmonaire et abdomino-pelvien confortant le diagnostic de xanthoma disséminatum. L´atteinte médiatino-pulmonaire s´est compliquée de pneumopathie dyspnéisante n´ayant pas régressé sous prednisone à 1mg/Kg/jour. Le décès fut noté en 2019 dans un tableau de détresse respiratoire, 5 mois après une chirurgie d´exérèse d´une volumineuse masse médiastinale qui comprimait les organes. La difficulté diagnostic des histiocytoses non langerhansiennes dans notre contexte d´exercice est liée en grande partie à l´inaccessibilité de l´immunohistochimie. En effet, si nous avions disposé assez tôt du marquage cellulaire positif au CD68 et négatif auCD1a, le diagnostic serait assez précoce. Ainsi, nous pourrions proposer des traitements pour améliorer la qualité de vie de l´enfant.

**Figure 1:**
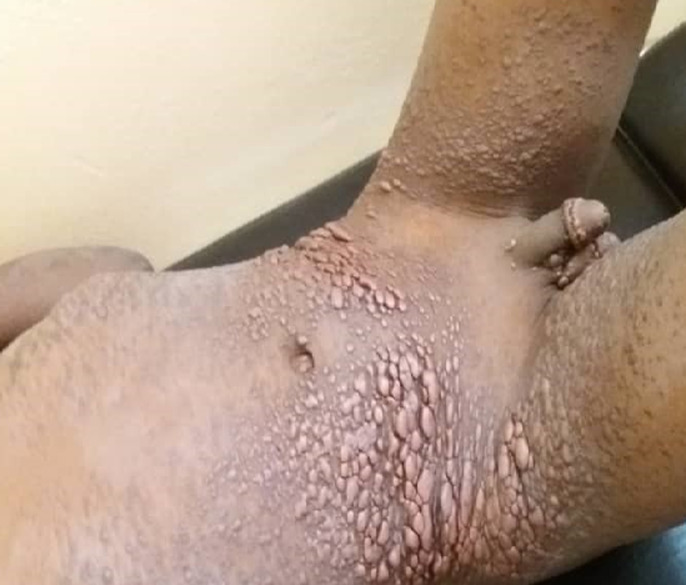
lésions cutanées papulo-nodulaire orangées diffuses de xanthoma disséminatum

